# Anti-Inflammatory Effects of β_2_-Receptor Agonists Salbutamol and Terbutaline Are Mediated by MKP-1

**DOI:** 10.1371/journal.pone.0148144

**Published:** 2016-02-05

**Authors:** Tiina Keränen, Tuija Hömmö, Mari Hämäläinen, Eeva Moilanen, Riku Korhonen

**Affiliations:** The Immunopharmacology Research Group, University of Tampere School of Medicine, and Tampere University Hospital, Tampere, Finland; French National Centre for Scientific Research, FRANCE

## Abstract

Mitogen-activated protein kinase phosphatase 1 (MKP-1) expression is induced by inflammatory factors, and it is an endogenous suppressor of inflammatory response. MKP-1 expression is increased by PDE4 inhibitor rolipram suggesting that it is regulated by cAMP-enhancing compounds. Therefore, we investigated the effect of β_2_-receptor agonists on MKP-1 expression and inflammatory response. We found that β_2_-receptor agonists salbutamol and terbutaline, as well as 8-Br-cAMP, increased MKP-1 expression. Salbutamol and terbutaline also inhibited p38 MAPK phosphorylation and TNF production in J774 mouse macrophages. Interestingly, salbutamol suppressed carrageenan-induced paw inflammation in wild-type mice, but the effect was attenuated in MKP-1(-/-) mice. In conclusion, these data show that β_2_-receptor agonists increase MKP-1 expression, which seems to mediate, at least partly, the observed anti-inflammatory effects of β_2_-receptor agonists.

## Introduction

Mitogen-activated protein kinases (MAPKs) are important intracellular signaling pathways that regulate many physiological and pathophysiological events in cells. The three main MAPK pathways include p38 MAPK, Jun N-terminal kinase (JNK) and extracellular signal-regulated kinase (ERK) [1,2]. MAPK pathways are three-tier kinase cascades that are activated in response to several extracellular signals, such as cytokines, growth factors and bacterial substances through G-protein-coupled and/or kinase-linked receptors. Upon activation, threonine and tyrosine residues in the activation motif of the given MAPK are phosphorylated by the upstream kinase in the signaling cascade [3,4]. Targets of activated MAPKs include transcription factors and other regulatory proteins, and they regulate many physiological cellular processes, such as cell growth, proliferation, differentiation, motility, stress response, survival, and apoptosis [1,2]. p38 MAPK and JNK have also a marked role in inflammation and immune response. They regulate the production of inflammatory cytokines, such as tumor necrosis factor (TNF), interleukin-6 (IL-6) and other mediators, such as prostaglandins and nitric oxide. Also, p38 MAPK and JNK augment Th1 type immune response and support the activation and functions of Th1 cells [1,5,6].

Dual specificity phosphatases (DUSPs) are endogenous factors that dephosphorylate tyrosine and threonine residues of their target proteins. Mitogen-activated protein kinase phosphatases (MKPs) are a subgroup of DUSPs, and they specifically dephosphorylate MAPKs, which makes them endogenous suppressors of activated MAPK pathways. MKP family of phosphatases has eleven members, and they display differences in substrate specificity among MAPKs, as well as in tissue distribution, cellular location and expressional pattern [7,8]. MAP kinase phosphatase 1 (MKP-1) is a nuclear phosphatase and it regulates p38 MAPK, and in some cells, JNK activity [6,9]. It has earlier been shown that hypoxia and inflammatory signals increase MKP-1 expression [10], and by inhibiting p38 MAPK, MKP-1 suppresses inflammatory gene expression and attenuates inflammatory response [11,12]. Interestingly, MKP-1 has also been reported to mediate certain anti-inflammatory drug effects. MKP-1 expression is increased by glucocorticoids and anti-rheumatic gold-compounds, and MKP-1 mediates, in part, the anti-inflammatory effects of these drugs [13,14]. Recently, we demonstrated that phosphodiesterase (PDE) 4 inhibitor rolipram increased MKP-1 levels and suppressed inflammatory response in wild-type mice, but the response was impaired in MKP-1(-/-) mice [15].

Salbutamol and terbutaline are β_2_-receptor agonists used in the treatment of obstructive lung diseases as bronchodilating remedy. β_2_-receptors are G protein-coupled receptors and their activation stimulates G_s_-proteins leading to increased adenylate cyclase activity and elevation of cAMP levels in cells [16–18]. In addition to their bronchodilation effects, β_2_-receptor agonists have been shown to possess certain anti-inflammatory properties in immune and inflammatory cells, which effects may contribute to the therapeutic drug effects in the treatment of inflammatory lung diseases. In experimental acute lung injury, β_2_-receptor agonists have been reported for example to attenuate proinflammatory activity and neutrophil recruitment. Combinations of β_2_-receptor agonists’ bronchodilatory and anti-inflammatory properties improve the value of these drugs in the treatment of acute and chronic lung diseases [19]. Because MKP-1 promotor contains a cAMP response element CRE [20,21], we hypothesized that cAMP elevating β_2_-receptor agonists may regulate the expression of this important endogenous anti-inflammatory factor. In the present study we investigated the effects of salbutamol on MKP-1 expression and further, whether MKP-1 is involved in the anti-inflammatory effects of this β_2_-receptor agonist.

## Methods

### Materials

Reagents were obtained as follows. Salbutamol [α-((*tert*-butylamino)methyl)-4-hydroxy-*m*-xylene-α,α´-diol], terbutaline [5-(2-(*tert*-butylamino)-1-hydroxyethyl)benzene-1,3-diol], 8-Br-cAMP (8-bromoadenosine 3´,5´-cyclic monophosphate) and LPS from *Escherichia coli* strain 0111:B4 were purchased from Sigma-Aldrich Inc. (St. Louis, MO, USA). Rolipram [4-(3-cyclopentyloxy-4-methoxy-phenyl)-pyrrolidin-2-one] and BIRB 769 [1-(5-*tert*-butyl-2-*p*-tolyl-2H-pyrazol-3-yl)-3(4-(2-morpholin-4-yl-ethoxy)naphthalen-1-yl)urea] were obtained from Axon Medchem BV (Groningen, the Netherlands). All other reagents were purchased also from Sigma-Aldrich Inc. (St. Louis, MO, USA) unless otherwise stated below.

### Cell culture

J774 mouse macrophages (ATCC, Rockville Pike, MD, USA) were cultured at +37°C in 5% CO_2_ atmosphere in Dulbecco´s Modified Eagle´s Medium supplemented with glutamax-1 (DMEM; Invitrogen, Paisley, UK) containing 10% (v/v) heat-inactivated FBS (fetal bovine serum), 100 U/ml penicillin, 100 μg/ml streptomycin and 250 ng/ml amphotericin B (all from Gibco, Wien, Austria). For experiments, cells (2.5 x 10^5^ cells/well) were seeded on 24-well plates and the cell monolayers were grown for 72 h before the experiments were started.

Salbutamol, terbutaline and rolipram were dissolved in dimethyl sulfoxide (DMSO), and 8-Br-cAMP and LPS in phosphate buffered saline (PBS). LPS (10 ng/ml) and/or the compounds of interest at the concentrations indicated or the solvent (DMSO, 0.1% v/v) were added to the cells in fresh culture medium containing 10% FBS and the supplements. Cells were further incubated for the time indicated.

The effect of LPS and the tested chemicals on cell viability was evaluated by modified XTT test (Cell Proliferation Kit II; Roche Diagnostics, Mannheim, Germany). Neither LPS nor the other chemicals used in the experiments were observed to evoke cytotoxicity.

### Preparation of cell lysates and Western blot analysis

At the indicated time points, culture medium was removed. Cells were rapidly washed with ice-cold phosphate-buffered saline (PBS) and solubilized in cold lysis buffer containing 10 mM Tris-HCl, 5 mM EDTA, 50 mM NaCl, 1% Triton X-100, 0.5 mM phenylmethylsulfonyl fluoride, 1 mM sodiumorthovanadate, 20 μg/ml leupeptin, 50 μg/ml aprotin, 5 mM sodium fluoride, 2 mM sodium pyrophosphate and 10 μM *n*-octyl-β-D-glucopyranoside. After incubation for 20 min on ice, lysates were centrifuged (12 000 g, 10 min, +4°C) and supernatants were collected and mixed in a ratio 1:4 with SDS loading buffer (62.5 mM Tris-HCl, pH 6.8, 10% glycerol, 2% SDS, 0.025% bromophenol blue and 5% β-mercaptoethanol) and stored at -20°C until analyzed. Protein concentrations in the samples were measures by the Coomassie Brilliant Blue method (Coomassie Protein Assay Reagent Kit; Pierce, Rockford, IL, USA).

Before Western blot analysis, the samples were boiled for 10 min. Equal aliquots of protein (20 μg) were loaded on 12% SDS-polyacrylamide gels and separated by electrophoresis. Proteins were transferred to Hybond enhance chemiluminescence nitrocellulose membrane (Amersham Biosciences, Buckinghamshire, UK) by semi-dry electroblotting. After transfer the membrane was blocked in TBS/T [20 mM Tris-base (pH 7.6), 150 mM NaCl, 0.1% Tween-20] containing 5% non-fat milk for 1 h at room temperature. For detection of phosphorylated proteins, membranes were blocked in TBS/T containing 5% bovine serum albumin (BSA). Membranes were incubated overnight at +4°C with the primary antibody and at room temperature for 1 h with the secondary antibody, and the chemiluminescent signal was detected by ImageQuant™ LAS 4000 mini (GE Healthcare Bio-Sciences AB, Uppsala, Sweden). The chemiluminescent signal was quantified with FluoChem program (version 3.1) and Image Quant TL 7.0 Image Analysis software.

Following antibodies were used in the Western blot analysis: MKP-1 (SAB2500331; Sigma-Aldrich Inc., St. Louis, MO, USA), p38 MAPK (ab27986; Abcam, Cambridge, UK) and phospho-p38 MAPK (#9211; Cell Signaling Technology Inc., Beverly, MA, USA), as well as actin (sc-1615), polyclonal anti-goat (sc-2020) and polyclonal anti-rabbit (sc-2004) (all three from Santa Cruz Biotechnology, Santa Cruz, CA, USA).

### RNA extraction and quantitative real-time reverse transcription polymerase chain reaction (qRT-PCR)

At the indicated time points, the culture medium was removed, and cell homogenization and total RNA extraction was carried out by using GenElute^TM^ Mammalian Total RNA Miniprep Kit (Sigma-Aldrich Inc., St. Louis, MO, USA) according to the manufacturer´s instructions. Reverse transcription of RNA to cDNA was performed by TaqMan^®^ Reverse Transcription Reagent kit (Applied Biosystems, Foster City, CA, USA), according to the supplier´s instructions. The primer and probe sequences and concentrations were optimized according to the manufacturer´s guidelines in TagMan^®^ Universal PCR Master Mix Protocol part number 4304449 revision C (Applied Biosystems, Branchburg, NJ, USA). The following primer and probe sequences were used: for mouse MKP-1 5´-AAGGATGCTGGAGGGAGAGT-3´ (forward), 5´-TGAGGTAAGCAAGGCAGATGGT-3´ (reverse) and 5´-TTTGTTCATTGCCAGGCCGGCAT-3´ (probe containing 6-FAM as the 5´-reporter dye and TAMRA as the 3´-quencher); for mouse TNF 5´-AATGGCCTCCCTCTCATCAGTT-3´ (forward), 5´-TCCTCCACTTGGTGGTTTGC-3´ (reverse) and 5´-CTCAAAATTCGAGTGACAAGCCTGTAGCCC-3´ (probe containing 6-FAM as the 5´-reporter dye and TAMRA as the 3´-quencher); for mouse GAPDH 5´-GCATGGCCGGCCGTGTTC-3´ (forward), 5´-GATGTCATCATACTTGGCAGGTTT-3´ (reverse) and 5´- TCGTGGATCTGACGTGCCGCC-3´ (probe containing 6-FAM as the 5´-reporter dye and TAMRA as the 3´-quencher). Primers and probes were obtained from Metabion (Martinsried, Germany).

PCR reaction parameters were as follows: incubation at +50°C for 2 min and at +95°C for 10 min, and thereafter 40 cycles of denaturation at +95°C for 15 s and annealing and extension at +60°C for 1 min. Each sample was determined in duplicate. A standard curve method was used to estimate the relative mRNA levels. When calculating the results, MKP-1 and TNF mRNA levels were first normalized against GAPDH.

### Enzyme-Linked Immunosorbent Assay (ELISA)

Culture medium samples and cell lysates were kept at -20°C until assayed. The concentrations of mouse TNF (Duoset^®^ ELISA Development System mouse TNF kit; R&D Systems Europe Ltd., Abindgon, UK) and mouse cAMP (cAMP ELISA Kit; Cell Biolabs, Inc. San Diego, CA, USA) were determined by ELISA according to the manufacturer´s instructions.

### Animals

Carrageenan-induced paw edema was carried out in wild-type and MKP-1(-/-) C57BL/6 mice. The MKP-1 deficient mice were originally generated in the laboratory of R. Bravo at Bristol-Myers Squibb Pharmaceutical Research Institute (Princeton, NJ, USA) and those as well as corresponding wild-type mice were bred at the University of Tampere School of Medicine animal facilities under conditions of optimum light (12:12 light-dark cycle), temperature (+22 ± 1°C) and humidity (50–60%), and food and water provided *ad libitum*. The study was approved by the National Animal Experiment Board. Female mice aged 10–12 weeks were used in the study.

### Carrageenan-induced paw edema

MKP-1 deficient and wild-type C57BL/6 mice (20–25 g) were divided into groups of six mice and treated with 200 μl of PBS or salbutamol (5 mg/kg in PBS) [22,23] by an i.p. injection 2 h before applying carrageenan. Before the administration of carrageenan, the mice were anaesthetized by i.p. injection of 0.5 mg/kg of medetomidine (Domitor^®^ 1 mg/ml; Orion Oyj, Espoo, Finland) and 75 mg/kg of ketamine (Ketalar^®^ 10 mg/ml; Pfizer Oy Animal Health, Helsinki, Finland). The mice received a 45 μl i.d injection of λ-carrageenan (2% dissolved in normal saline) in one hind paw. The contralateral paw received 45 μl of saline and it was used as a control. Paw volume was measured before and 2 h, 4 h and 6 h after the carrageenan injection with a plethysmometer (Ugo Basile, Comerio, Italy). Edema is expressed as the difference between the volume changes of the carrageenan-treated paw and the control paw.

### Statistics

Results are expressed as mean ± standard error of mean (S.E.M.). When appropriate, Student´s t-test, one-way ANOVA with Dunnett´s or Bonferroni´s post test or two-way ANOVA with Bonferroni´s post test was performed using GraphPad Prism-5 version 5.04 for Window XP (GraphPad Software Inc., La Jolla, CA, USA). P values less than 0.05 were considered significant.

## Results

### β_2_-receptor agonists salbutamol and terbutaline enhanced MKP-1 expression in activated mouse macrophages

**Fig 1:**
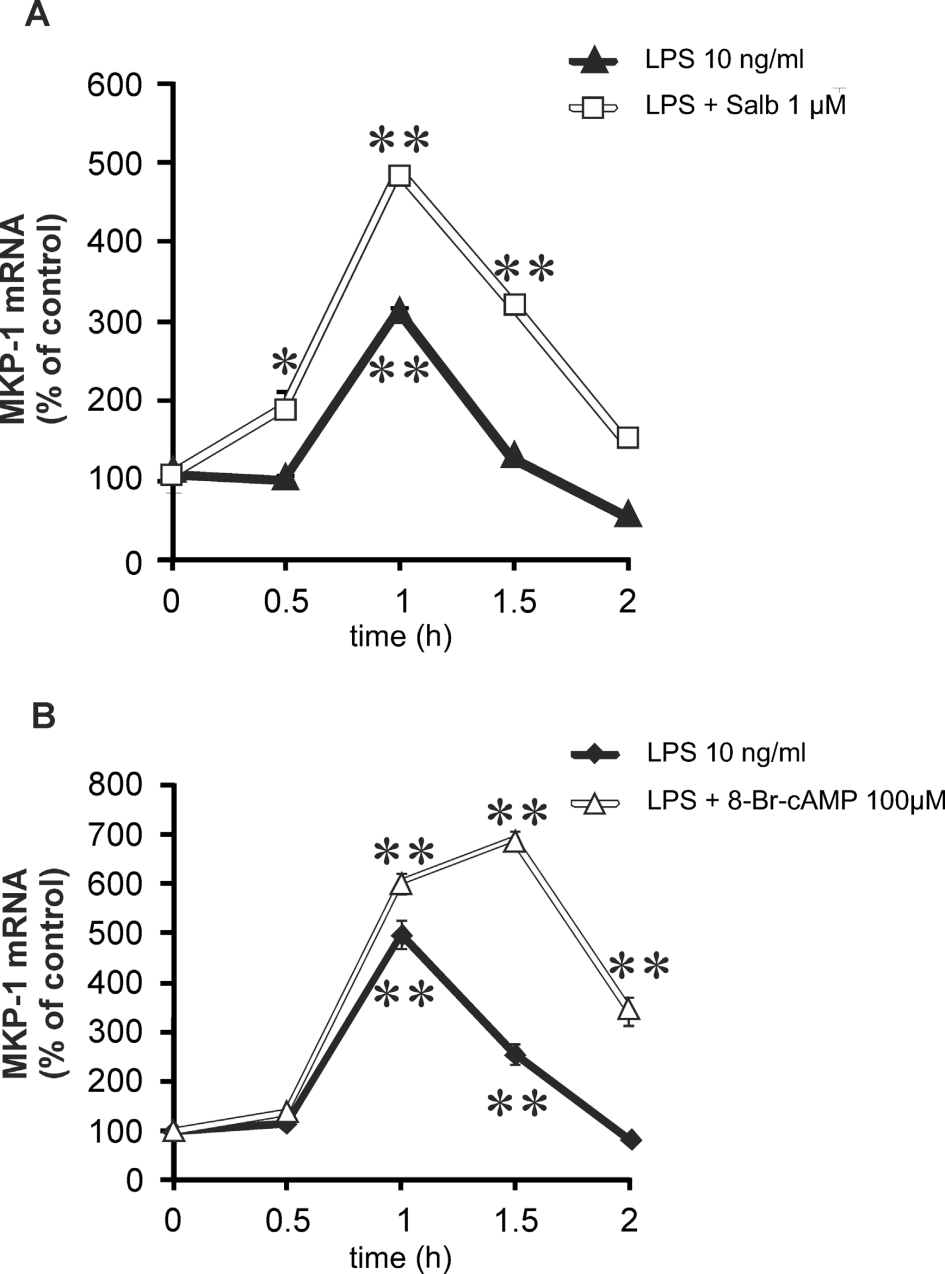
Salbutamol and cAMP analog 8-Br-cAMP enhanced MKP-1 expression in J774 macrophages. J774 macrophages were stimulated with LPS (10 ng/ml) in the presence or in the absence of salbutamol (A) or 8-Br-cAMP (B) for the time indicated. MKP-1 mRNA was measured by quantitative RT-PCR, and MKP-1 mRNA expression levels were normalized against GAPDH mRNA levels. Results are expressed as mean ± S.E.M., n = 3. One-way ANOVA with Dunnett´s post test was performed, and statistical significance is indicated with * p < 0.05 and ** p < 0.01 as compared to control cells.

**Fig 2:**
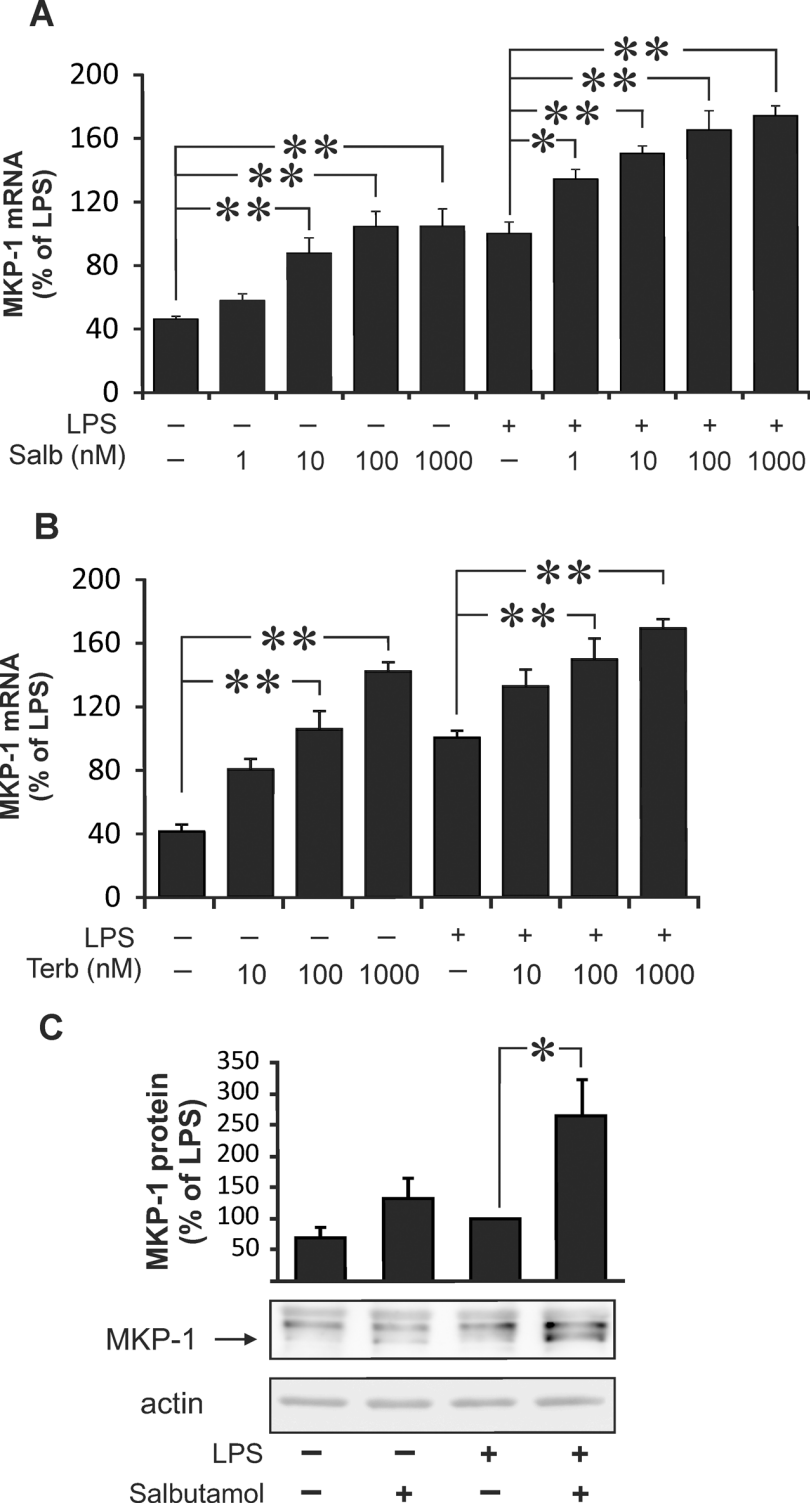
Salbutamol and terbutaline enhanced MKP-1 expression in J774 macrophages in a dose-dependent manner. (A and B) J774 macrophages were treated with increasing concentrations of salbutamol or terbutaline in the absence or in the presence of LPS (10 ng/ml) for 1 h. MKP-1 mRNA was measured by quantitative RT-PCR, and MKP-1 mRNA expression levels were normalized against GAPDH mRNA levels. (C) J774 cells were incubated with LPS (10 ng/ml) and salbutamol (100 nM) for 1 hour and MKP-1 protein was measured by Western blot. The chemiluminescent signal was quantified, and the amounts of MKP-1 were normalized against actin. Results are expressed as mean ± S.E.M., n = 6 (A and B) or n = 4 (C). One-way ANOVA with Dunnett´s (A and B) or Bonferroni´s (C) post test was performed, and statistical significance is indicated with * p < 0.05, ** p < 0.01 and *** p < 0.001 as compared to control or LPS-treated cells.

**Fig 3:**
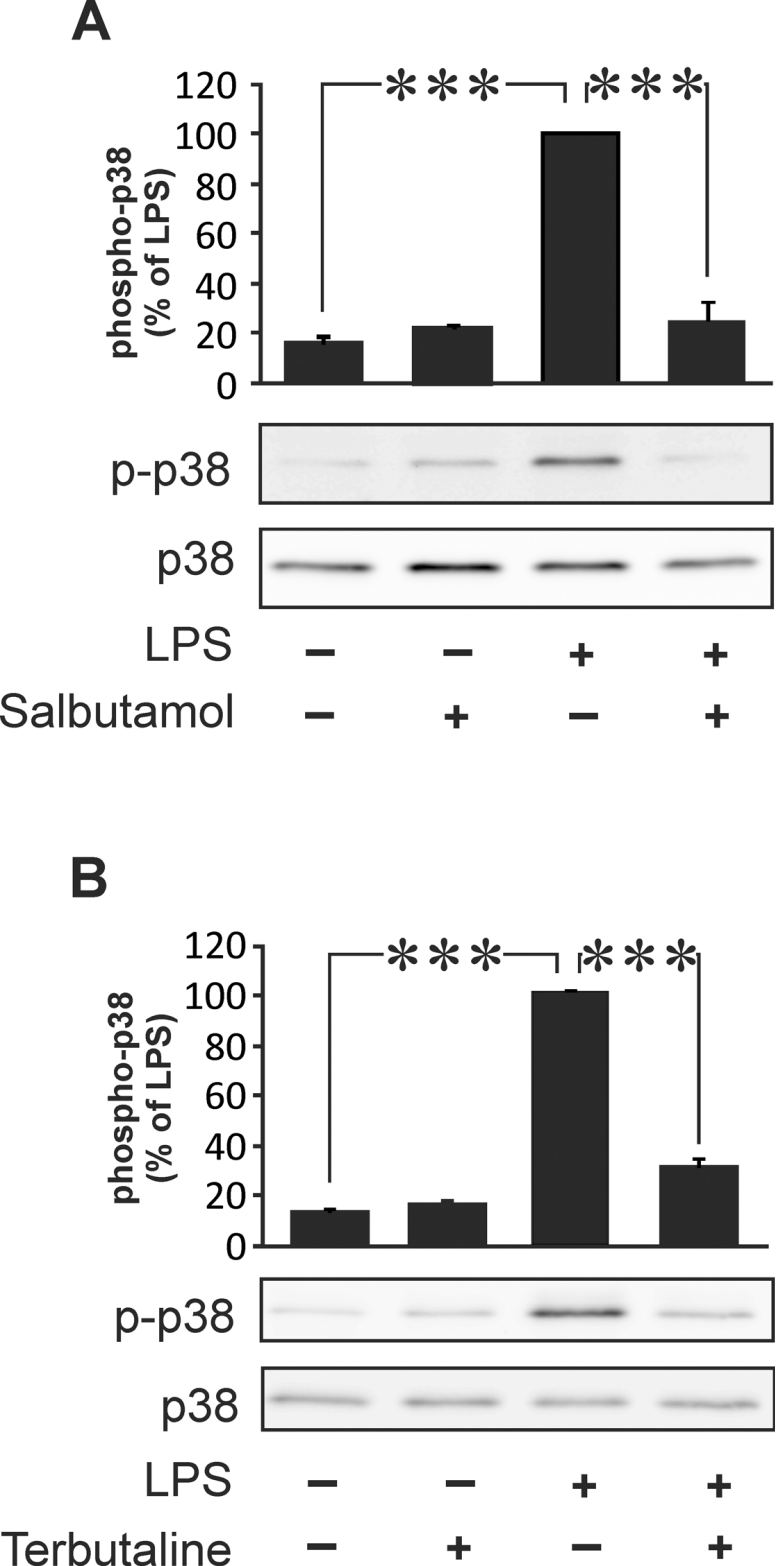
Salbutamol and terbutaline reduced the phosphorylation of p38 MAPK in J774 macrophages. J774 macrophages were stimulated with LPS (10 ng/ml) in the absence or in the presence of salbutamol (100 nM, A) or terbutaline (100 nM, B) for 1 h, and the phosphorylation of p38 MAPK was detected by Western blot. The chemiluminescent signal was quantified, and phosphorylated p38 MAPK was normalized against total p38 MAPK. Results are expressed as mean ± S.E.M, n = 8. One-way ANOVA with Bonferroni´s post test was performed, and statistical significance is indicated with *** p < 0.001 as compared to LPS-treated cells.

MKP-1 promoter has been described to contain *cis*-regulator CRE sequences [20,21]. Therefore we hypothesized that cAMP-elevating compounds would regulate MKP-1 expression. As expected, β_2_-receptor agonist salbutamol increased intracellular cAMP levels in J774 macrophages (Table 1). Next, we investigated the effects of salbutamol on MKP-1 expression in these cells. MKP-1 mRNA expression was increased by LPS and, interestingly, it was further enhanced by salbutamol and by a cAMP analog 8-Br-cAMP (Fig 1). Salbutamol and another β_2_-receptor agonist terbutaline increased MKP-1 expression alone and in combination with LPS in J774 macrophages in a dose-dependent manner (Fig 2). MKP-1 is an endogenous suppressor of p38 MAPK activity. Therefore, we investigated the effect of β_2_-receptor agonists salbutamol and terbutaline on p38 MAPK phosphorylation. The phosphorylation of p38 MAPK was increased in response to LPS and it was inhibited by β_2_-receptor agonists in J774 macrophages (Fig 3).

**Table 1:** Salbutamol increased intracellular cAMP levels in J774 macrophages.

treatment	cAMP (pg/ml)	
Control	1.08 ± 0.8	
Salb 100 nM	1230.9 ± 251.8	a
LPS 10 ng/ml	20.6 ± 11.1	
LPS+ Salb	730.5 ± 246.5	b

J774 macrophages were incubated with salbutamol (Salb) and stimulated with LPS for 1 min. Cells were then lysed and cAMP levels were measured by ELISA. Results are expressed as mean ± S.E.M, n = 5. One-way ANOVA with Bonferroni´s post test was performed, and statistical significance is (a) p < 0.001 between untreated cells and cells treated with salbutamol and (b) p < 0.001 between LPS-treated cells and cells treated with the combination of LPS and salbutamol.

### β_2_-receptor agonists salbutamol and terbutaline inhibited TNF production in activated mouse macrophages

**Fig 4:**
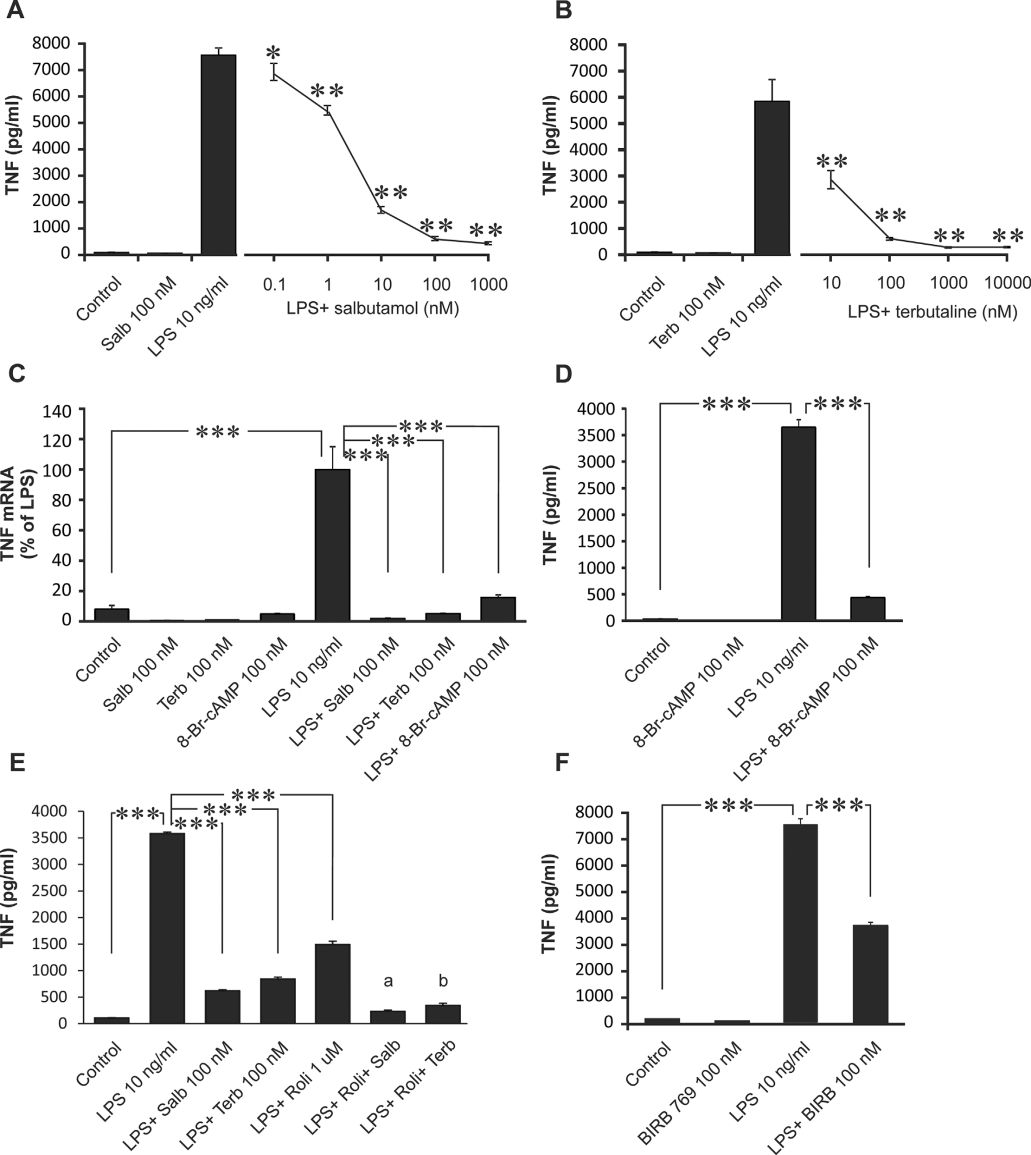
Salbutamol, terbutaline, 8-Br-cAMP and the p38 MAPK inhibitor BIRB 769 inhibited TNF production in J774 macrophages. J774 macrophages were stimulated with LPS (10 ng/ml) in the absence or in the presence of salbutamol (A, C and E), terbutaline (B, C and E), 8-Br-cAMP (C and D), rolipram (E) or the p38 MAPK inhibitor BIRB 769 (F) for 24 h (A, B, D, E and F; TNF protein) or for 4 h (C; TNF mRNA). TNF mRNA was measured by quantitative RT-PCR and TNF mRNA expression levels were normalized against GAPDH mRNA levels. TNF protein accumulation in the culture medium was measured by ELISA. Results are expressed as mean ± S.E.M., n = 8 (A-C) or n = 4 (D, E and F). One-way ANOVA with Dunnett´s post test (A and B) or Bonferroni´s post test (C-F) was performed and statistical significance is indicated with * p < 0.05, ** p < 0.01 and *** p < 0.001 as compared to LPS-treated cells. In the Panel 4E, statistical significance (a) is p < 0.001 between LPS+ salbutamol and LPS+ salbutamol+ rolipram treated cells, and (b) is p < 0.001 between LPS+ terbutaline and LPS+ terbutaline+ rolipram treated cells.

TNF is a cytokine whose expression is known to be regulated by MKP-1 and p38 MAPK [15,24]. Therefore, we continued by investigating the effects of β_2_-receptor agonists on TNF production. Salbutamol and terbutaline as well as cAMP analog 8-Br-cAMP inhibited LPS-induced TNF mRNA and protein expression, and maximal/submaximal inhibition of TNF protein release was observed with 100 nM drug concentration in J774 macrophages (Fig 4). TNF production was further inhibited when salbutamol or terbutaline was combined with a PDE4 inhibitor rolipram (Fig 4E). We also investigated the effect of p38 MAPK inhibitor on TNF production. As expected, p38 MAPK inhibitor BIRB 796 inhibited LPS-induced TNF release in macrophages (Fig 4F).

### The inhibition of carrageenan-induced paw inflammation by salbutamol was mediated by MKP-1

**Fig 5:**
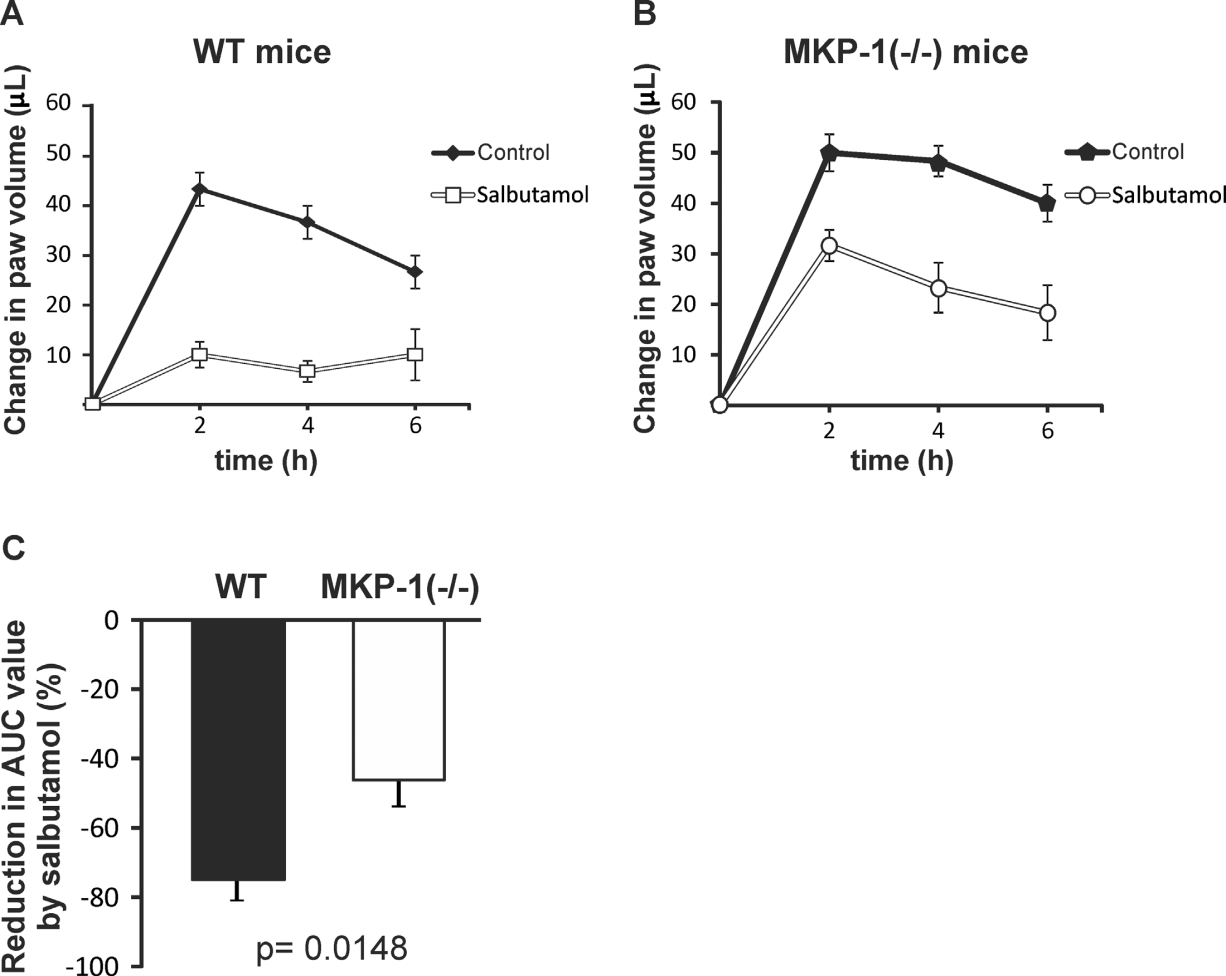
Salbutamol attenuated carrageenan-induced paw inflammation in mice. Mice were treated with salbutamol (5 mg/kg, i.p.) 2 h before the experiment. In the beginning of the experiment (0 h), hind paw volumes were measured with a plethysmometer. After that, carrageenan (2%) or vehicle (control) was injected into the paw and the paw edema was measured with plethysmometer at the time points indicated. Edema is expressed as the difference between the volume changes of the carrageenan-treated paw and the control paw. Mean ± S.E.M., n = 6. The Fig 5C presents the percent decrease in AUC values by salbutamol.

As salbutamol increased MKP-1 expression, we wanted to investigate whether MKP-1 could mediate the anti-inflammatory effects of salbutamol, therefore we tested the effect of salbutamol on the severity of carrageenan-induced paw inflammation in wild-type and MKP-1(-/-) mice. Carrageenan-induced paw edema was increased in MKP-1(-/-) mice as compared to wild-type mice (AUC values were 186.7 ± 13.1 μL/h and 236.7 ± 15.9 μL/h in WT and MKP-1(-/-) mice, respectively, p = 0.0352, n = 6, Fig 5). Carrageenan-induced paw edema was clearly attenuated by salbutamol in wild-type mice (73% reduction in AUC value), while salbutamol was less effective to reduce paw edema in MKP-1(-/-) mice (43% reduction in AUC value, p = 0,0148) (Fig 5).

## Discussion

The aim of the present study was to investigate the effects of β_2_-receptor agonist salbutamol on MKP-1 expression, on TNF production and on acute inflammatory response *in vivo*. We found that salbutamol increased MKP-1 expression, inhibited the phosphorylation of p38 MAPK and suppressed the production of TNF. We used another β_2_-receptor agonist terbutaline as a reference compounds and its effects on p38 MAPK phosphorylation and TNF production were comparable to those of salbutamol. Salbutamol suppressed carrageenan-induced acute inflammation *in vivo* in wild-type mice, but that effect was attenuated in MKP-1(-/-) mice in a statistically significant manner. These results suggests that acute anti-inflammatory effects of β_2_-receptor agonists are partly mediated by MKP-1.

β_2_-receptor agonists are used as a bronchodilators in obstructive lung diseases [17,19]. β_2_-receptor agonists have been reported to have anti-inflammatory effects in addition to their effects on smooth muscle relaxation in the airways. They have been shown to inhibit the expression of inflammatory mediators and to reduce capillary permeability and formation of plasma exudate and tissue edema [25,26]. Salbutamol also reduced carrageenan-induced paw edema in rats and that effect was attenuated when the β_2_-receptors were blocked by a non-selective β-receptor antagonist propranolol [27]. In this study, we found that β_2_-receptor agonists salbutamol and terbutaline inhibited the production of TNF in macrophages. We also found that carrageenan-induced paw edema was reduced by salbutamol, which is in line with the previous studies in rats [27]. These results show that β_2_-receptor agonists have anti-inflammatory effects *in vitro* and *in vivo*, and these findings are in line with the previously published reports.

MKPs belong to a larger family of DUSPs, and they are endogenous suppressors of MAPK activity [28,29]. MKPs control MAPK activity by dephosphorylating the activation domain (Thr-X-Tyr, threonine and tyrosine residues) of MAPK, which inactivates the kinase. MKPs regulate the inflammatory response as well as other cellular functions including apoptosis, cell cycle, proliferation and differentiation [7,30].

MKP-1 is an interesting member of the MKP phosphatase family. MKP-1 is expressed in most cell types and tissues in human body. It is a nuclear tyrosine/threonine phosphatase that regulates primarily the activity of p38 MAPK, and in some cells, JNK [5,31]. p38 MAPK pathway is an important regulator of inflammation and immune response [2]. p38 MAPK inhibitors attenuate the production of inflammatory mediators [11,31–33], and they have clear anti-inflammatory effects in experimental models of inflammatory diseases [34–36]. Studies with MKP-1(-/-) mice have shown that MKP-1 suppresses inflammatory gene expression (such as TNF and IL-6), and attenuates acute and chronic inflammatory response by inhibiting p38 MAPK [11,12,31,37]. Despite excessive inflammatory response, MKP-1(-/-) mice display defective anti-microbial responses due to reduced IL-12 production, impaired Th1 response and excessive IL-10 release [38–40]. Interestingly, in ovalbumin-induced airway inflammation model, a common experimental asthma model, the increased activation of p38 MAPK coincide with the decreased expression of MKP-1 [41].

cAMP is an important intracellular second messenger that mediates many effects of β_2_-receptor agonists. MKP-1 promoter contains two binding sites for the transcription factor cAMP responsive element binding protein (CREB) [20]. We found that salbutamol enhanced cAMP level in macrophages, and that cAMP analog 8-Br-cAMP enhanced MKP-1 expression as did β_2_-receptor agonists. cAMP has been reported to increase the expression of MKP-1 by activating protein kinase A-CREB pathway [15,42–45].

Suppression of IL-8 production by β_2_-agonists has previously been shown to occur concomitantly with increased MKP-1 expression [46,47]. Accordingly, we found here that salbutamol suppressed TNF production in macrophages along with increased MKP-1 expression and decreased p38 MAPK phosphorylation. More interestingly, the current findings extend the previous data by providing *in vivo* evidence that MKP-1 mediates the anti-inflammatory effects of salbutamol, at least partly. Carrageenan-induced acute inflammatory response was inhibited by salbutamol in wild-type mice, but that effect was impaired in MKP1(-/-) mice. This strongly suggests that MKP-1 participates in the anti-inflammatory effects of β_2_-receptor agonists. In this study, we found that β_2_-agonists were more potent inhibitors of TNF production as compared to that seen with p38 MAPK inhibitor. This suggests that the anti-inflammatory effects of β_2_-agonists are mediated not only through inhibition of p38 MAPK activity by MKP-1 but there are other anti-inflammatory mechanisms involved, also. This is also supported by the finding showing that the inhibition of carrageenan-induced inflammatory response by salbutamol was partially but not completely, impaired in MKP-1(-/-) mice. For instance, cAMP has been reported to inhibit macrophage phagocytosis through a cAMP effector Exchange protein activated by cAMP-1 [48].

Importantly, MKP-1 is linked to certain other important anti-inflammatory drug effects. The expression of MKP-1 is increased by glucocorticoids, and MKP-1 mediates, at least partly, the anti-inflammatory effects of glucocorticoids [13,49]. Earlier it has been reported that long-acting β_2-_agonists increased MKP-1 levels in airway smooth muscle cells [50].We have shown that disease-modifying anti-rheumatic gold compounds enhance MKP-1 expression along with their inhibitory effects on the production of IL-6, cyclooxygenase-2 and matrix metalloproteinase 3, and those effects were mediated by MKP-1 [14]. Recently, we have also demonstrated that a PDE4 inhibitor rolipram increased MKP-1 levels and suppressed inflammatory response in wild-type mice, but the inhibition of the inflammatory response was severely impaired in MKP-1(-/-) mice [15]. Interestingly, we found here that combining rolipram to β_2_-agonist further inhibited TNF production when compared to that with β_2_-agonist alone. This is interesting because PDE4 inhibitor roflumilast is used as an anti-inflammatory remedy in COPD and it increases cAMP levels by abrogating the enzymatic degradation of cAMP to 5’AMP [46,51,52]. This further supports the idea that the anti-inflammatory effects of β_2_-agonists are mediated by cAMP and that combination of β_2_-agonist and PDE4 inhibitor would have improved anti-inflammatory effect through enhanced MKP-1 expression.

Hence, MKP-1 is not only an endogenous suppressor of inflammatory response, but it also seems to mediate therapeutic effects of certain anti-inflammatory drugs. Even though β_2_-receptor agonists are primarily used as bronchodilators, they may also have anti-inflammatory effects [19]. Increased MKP-1 expression could be a significant mechanism mediating the anti-inflammatory effects of β_2_-receptor agonists. The present findings further emphasize the potential of MKP-1 as a novel anti-inflammatory drug target, and its significance in the pathophysiology and treatment of airway inflammation.

In conclusion, we found that β_2_-receptor agonists increased MKP-1 expression and suppressed p38 MAPK phosphorylation (i.e. activity) as well as TNF production in macrophages. Importantly, MKP-1 mediated the anti-inflammatory effects of salbutamol *in vivo*, and this is a novel finding. The results presented here emphasize the importance of MKP-1 as a novel anti-inflammatory drug target.

## Abbreviations

COPD: chronic obstructive pulmonary disease
CRE: cAMP responsive element
CREB: cAMP responsive element binding protein
DUSP: dual-specificity phosphatase
ERK: extracellular-regulated kinase
IL: interleukin
LPS: lipopolysaccharide
JNK: Jun N-terminal kinase
MAPK: mitogen-activated protein kinase
MKP: mitogen-activated protein kinase phosphatase
PDE: phosphodiesterase
TNF: tumor necrosis factor
